# Molecular Characterization and Sterol Profiles Identify Nonsynonymous Mutations in *ERG2* as a Major Mechanism Conferring Reduced Susceptibility to Amphotericin B in Candida kefyr

**DOI:** 10.1128/spectrum.01474-23

**Published:** 2023-06-26

**Authors:** Mohammad Asadzadeh, Wadha Alfouzan, Josie E. Parker, Jacques F. Meis, Steven L. Kelly, Leena Joseph, Suhail Ahmad

**Affiliations:** a Department of Microbiology, Faculty of Medicine, Kuwait University, Safat, Kuwait; b Molecular Biosciences Division, School of Biosciences, Cardiff University, Cardiff, United Kingdom; c Department of Medical Microbiology and Infectious Diseases, Canisius-Wilhelmina Hospital, Nijmegen, the Netherlands; d Center of Expertise in Mycology, Radboudumc, Canisius-Wilhelmina Hospital, Nijmegen, the Netherlands; e Department of Internal Medicine, Excellence Center for Medical Mycology, Faculty of Medicine, University Hospital Cologne, Cologne, Germany; f Institute of Life Science, Faculty of Health, Medicine and Life Sciences, Swansea University, Swansea, Wales, United Kingdom; Institut Pasteur

**Keywords:** *Candida kefyr*, ERG2, nonsynonymous mutations, reduced susceptibility, amphotericin B

## Abstract

The molecular basis of reduced susceptibility to amphotericin B (rs-AMB) among any yeasts is poorly defined. Genetic alterations in genes involved in ergosterol biosynthesis and total cell sterols were investigated among clinical Candida kefyr isolates. C. kefyr isolates (*n* = 81) obtained from 74 patients in Kuwait and identified by phenotypic and molecular methods were analyzed. An Etest was initially used to identify isolates with rs-AMB. Specific mutations in *ERG2* and *ERG6* involved in ergosterol biosynthesis were detected by PCR sequencing. Twelve selected isolates were also tested by the SensiTitre Yeast One (SYO), and total cell sterols were evaluated by gas chromatography-mass spectrometry and *ERG3* and *ERG11* sequencing. Eight isolates from 8 patients showed rs-AMB by Etest, including 2 isolates with additional resistance to fluconazole or to all three antifungals. SYO correctly identified 8 of 8 rs-AMB isolates. A nonsynonymous mutation in *ERG2* was detected in 6 of 8 rs-AMB isolates but also in 3 of 73 isolates with a wild-type AMB pattern. One rs-AMB isolate contained a deletion (frameshift) mutation in *ERG2*. One or more nonsynonymous mutations was detected in *ERG6* in 11 of 81 isolates with the rs-AMB or wild-type AMB pattern. Among 12 selected isolates, 2 and 2 isolates contained a nonsynonymous mutation(s) in *ERG3* and *ERG11*, respectively. Ergosterol was undetectable in 7 of 8 rs-AMB isolates, and the total cell sterol profiles were consistent with loss of *ERG2* function in 6 rs-AMB isolates and loss of *ERG3* activity in another rs-AMB isolate. Our data showed that *ERG2* is a major target conferring rs-AMB in clinical C. kefyr isolates.

**IMPORTANCE** Some yeast species exhibit intrinsic resistance or rapidly acquire resistance to azole antifungals. Despite >50 years of clinical use, resistance to amphotericin B (AMB) among yeast species has been extremely rarely reported until recently. Reduced susceptibility to AMB (rs-AMB) among yeast species is, therefore, a matter of serious concern due to the availability of only four classes of antifungal drugs. Recent studies in Candida glabrata, Candida lusitaniae, and Candida auris have identified *ERG* genes involved in ergosterol biosynthesis as the major targets conferring rs-AMB. The results of this study also show that nonsynonymous mutations in *ERG2* impair its function, abolish ergosterol from C. kefyr, and confer rs-AMB. Thus, rapid detection of rs-AMB among clinical isolates will help in proper management of invasive C. kefyr infections.

## INTRODUCTION

The incidence of invasive fungal infections (IFIs) is rising globally due to increasing populations of immunocompromised or immunosuppressed individuals and other older hospitalized patients with multiple debilitating comorbidities (1–3). The IFIs occur more frequently among at-risk patients during their stay in intensive care units (ICUs) and are associated with high mortality rates (4–6). The spectrum of *Candida* and other yeast species causing IFIs is changing due to increasing use of antifungal prophylaxis (4, 7). Emergence of drug-resistant and multidrug-resistant (MDR) *Candida* and other yeast spp. is another matter of great concern due to the limited antifungal armamentarium available to treat invasive infections (6, 8–10). Recent reports have documented MDR among clinical isolates of Candida glabrata (11–13), Candida auris (14–16), Candida haemulonii complex members (17, 18), Candida lusitaniae (19), and Candida kefyr (20, 21).

Invasive rare yeast infections are difficult to treat due to their intrinsic or acquired resistance to one or more antifungal drugs, and clinical breakpoints are mostly unavailable (6, 22). Candida kefyr (Kluyveromyces marxianus) is a thermotolerant ascomycetous yeast that is isolated from diverse habitats (e.g., kefir grain, raw milk, fermented dairy products, sugar industry sewage, and plant material) and is also widely used in food and industrial biotechnology applications (23–25). Candida kefyr is now recognized as an emerging fungal pathogen among ICU patients, as infections due to this yeast are increasingly being reported in recent years in immunocompromised individuals particularly among patients with hematological malignancies (20, 22, 26–33). C. kefyr infections have been shown to occur more frequently during summer months among patients with hematologic malignancies (26). This phenomenon may be attributed to the consumption of dairy products containing overgrowth of C. kefyr as a result of lack of proper refrigeration during summertime. Indeed, consumption of dairy products (yogurt) containing C. kefyr has been shown recently by whole-genome sequencing studies as the most likely source of disseminated infection in an immunocompromised patient with mucositis during induction chemotherapy of acute myeloid leukemia (29). C. kefyr bloodstream infections are associated with higher mortality rates than C. albicans infections among ICU patients (2). Thus, proper patient management requires rapid diagnosis and accurate antifungal susceptibility testing (AFST) of clinical C. kefyr isolates. Recent studies have shown that clinical C. kefyr isolates exhibit reduced susceptibility to antifungals, particularly amphotericin B (20, 21, 28, 33). The molecular basis of reduced susceptibility to amphotericin B (rs-AMB) among *Candida* and yeast species, including C. kefyr, is poorly defined. A prevalence of <1% has recently been reported for C. kefyr among clinical yeast isolates, and some C. kefyr isolates exhibited rs-AMB in a previous study from Kuwait (20). Here, we describe genetic alterations in major genes involved in ergosterol biosynthesis and their association with total cell sterols among clinical C. kefyr, including rs-AMB isolates.

## RESULTS

### Clinical C. kefyr isolates and their susceptibility to antifungal drugs.

A total of 81 clinical C. kefyr isolates obtained from 74 patients (see Table S1 in the supplemental material) and identified by phenotypic and molecular methods were studied to decipher the molecular mechanisms conferring rs-AMB. The AFST data for AMB, fluconazole, voriconazole, and micafungin obtained by Etest are shown in Table 1. Based on epidemiological cutoff values described previously (34), 73 C. kefyr isolates were wild type (WT; minimum inhibitory concentration, MIC ≤ 1 μg/mL) while 8 isolates were non-WT (MIC > 1 μg/mL) for AMB (rs-AMB). On the contrary, based on the epidemiological cutoff values described previously for fluconazole, voriconazole, and micafungin (34, 35), only 2, 1, and 1 isolate were non-WT, while the remaining 79, 80, and 80 isolates were WT for fluconazole, voriconazole, and micafungin, respectively. One isolate (Kw2153/18) was non-WT for all four drugs, while another isolate (Kw135/15) was non-WT for AMB and fluconazole. When AFST was performed by using the Sensititre YeastOne (SYO) on 12 selected isolates, 10 isolates yielded concordant results for AMB, while two isolates WT by Etest scored as non-WT for AMB (rs-AMB). Surprisingly, isolate Kw2153/18, non-WT for AMB, fluconazole, voriconazole, and micafungin by Etest, was WT for fluconazole, voriconazole, and micafungin by SYO. Repeat testing of isolates with discrepant results yielded the same patterns by both Etest and SYO. The remaining isolates yielded concordant results for fluconazole, voriconazole, and micafungin by SYO (Table 2).

**TABLE 1:** MIC values of 81 C. kefyr isolates for amphotericin B, fluconazole, voriconazole, and micafungin by Etest

Antifungal	No. of isolates with MIC (μg/mL) of^*a*^:																				
drug	≤0.01	0.02	0.03	0.06	0.09	0.13	0.19	0.25	0.38	0.5	0.75	1	2	3	4	8	16	32	64	128	>256
Amphotericin B			3	6	1	2	8	12	21	11	6	3			**1**			**7**			
Fluconazole			1	1	3	3	19	13	10	17	7	5		**1**							**1**
Voriconazole	50	25	5															**1**			
Micafungin	3	22	15	30	7	2	1					**1**									

a The modal minimum inhibitory concentration (MIC) values are underlined. MIC values indicative of non-WT pattern for each antifungal drug are shown in boldface.

**TABLE 2:** Susceptibility to antifungal drugs by Etest and Sensititre YeastOne (SYO) methods, mutations in *ERG2*, *ERG3*, *ERG6*, and *ERG11* genes, and ergosterol contents^*a*^

Isolate		Etest MIC^*b*^ (μg/mL)				SYO MIC^*b*^ (μg/mL)				Mutation(s) identified in^*c*^:				% ergosterol^*d*^
no.	Source	AMB	FLU	VOR	MFG	AMB	FLU	VOR	MFG	*ERG2*	*ERG3*	*ERG6*	*ERG11*	
ATCC 28838	Human	0.25	0.09	≤0.01	0.03	1	0.25	≤0.01	0.03	A113S	WT	WT	WT	70.5 ± 7.4
Kw 1672/11	Urine	0.25	0.19	0.01	0.03	**2**	0.5	0.02	0.06	WT	WT	WT	WT	85.2 ± 0.9
Kw197/13	Sputum	0.19	0.5	≤0.01	0.03	0.5	0.25	≤0.01	0.03	A113S	WT	D168G	WT	64.0 ± 2.3
Kw 3153/14	Urine	0.13	0.38	≤0.01	0.05	0.5	≤0.12	≤0.01	0.02	WT	WT	D4E + K163Q	WT	85.9 ± 2.3
Kw 3267/17	Blood	0.19	0.5	≤0.01	0.05	**2**	0.5	≤0.01	0.06	A113S	WT	K163Q	WT	72.8 ± 5.7
Kw 3352/11	Urine	**32**	0.03	≤0.01	0.06	**8**	0.25	≤0.01	0.12	G121C	N313S	WT	WT	0
Kw135/15	Urine	**32**	**3**	0.03	0.09	**8**	**4**	0.03	0.25	E105K	WT	WT	K151R + Y227D	0
Kw 2327/17	BAL	**32**	0.5	0.02	0.09	**4**	≤0.12	≤0.01	0.06	M93I	WT	WT	WT	0
Kw 1075/18	Ear swab	**32**	0.5	0.03	0.09	**4**	1	0.03	0.25	H155R	WT	WT	WT	0
Kw 2153/18	ETT	**4**	**256**	**32**	**1**	**2**	0.5	0.03	0.06	WT	S218P	F363L	WT	0
Kw 1661/19	Sputum	**32**	0.75	0.03	0.06	**8**	0.5	0.02	0.25	Δnt 617t	WT	WT	WT	77.9 ± 0.3
Kw196-11/20	BAL	**32**	0.13	≤0.01	0.13	**2**	0.5	0.03	0.06	L107S	WT	WT	K189R	0
Kw20-12/20	Urine	**32**	0.25	0.02	0.19	**8**	0.5	0.02	0.25	G90C	WT	F363L	WT	0

a BAL, bronchoalveolar lavage; ETT, endotracheal secretion; AMB, amphotericin B; FLU, fluconazole; VOR, voriconazole; MFG, micafungin.

b The MIC values indicative of the non-WT pattern for each antifungal drug are shown in bold.

c The *ERG* gene sequences from C. kefyr ATCC 26548 were used as reference.

d Ergosterol values (percentages of total sterols) are means ± standard deviations for three replicates.

### Analysis of *ERG* gene sequences and ergosterol content.

The complete genome sequence data are available for 4 different C. kefyr strains, while susceptibility data are available for only 2 of these 4 strains. The encoded ERG2 protein (sterol C8-C7 isomerase) sequences are identical for C. kefyr ATCC 26548 WT for AMB (36) (GenBank accession number AP012218), C. kefyr 100656-19 WT for AMB (29) (GenBank accession number PRJEB33886), C. kefyr DMKU3-1042 (GenBank accession number NC_036030) and C. kefyr FIM1 (GenBank accession number CP015059). The DNA and encoded protein sequence data from C. kefyr ATCC 26548 were used as reference in this study. Compared to the reference sequence, several nonsynonymous mutations and one deletion frameshift mutation were detected among clinical C. kefyr isolates tested here. These included an Ala-to-Ser change at position 113 (A113S) mutation in 3 isolates WT for AMB as well as in C. kefyr ATCC 28838, which was used as a reference for sterol analyses in this study. Among 6 rs-AMB isolates, a nonsynonymous mutation (G90C, M93I, E105K, L107S, G121C, or H155R) was detected in 1 isolate each (Table 2). A G616A transition together with deletion of nucleotide T at position 617 (Δnt 617t), resulting in a frameshift at codon 206 and premature termination of the protein at codon 208 (KTV206ST*), was detected in 1 rs-AMB (Kw1661/19) isolate (Table 2). One rs-AMB isolate (Kw2153/18) and the remaining 70 isolates WT for AMB by Etest contained the wild-type sequence for *ERG2*; this also supported the use of the C. kefyr ATCC 26548 sequence as reference. A few synonymous mutations, including heterozygosity, were also detected among some isolates. Nonsynonymous mutations G90C, M93I, E105K, L107S, G121C, and H155R likely resulted in complete loss of ERG2 function, as ergosterol was totally absent among all 6 rs-AMB isolates with these mutations (Table 2). Furthermore, ergosta-type sterols accumulated, indicating the loss of ERG2 protein function in the isolates with these mutations (described in more detail below). No ergosterol was detected in rs-AMB isolate Kw2153/18 with WT sequence for the ERG2 protein, while nearly normal levels were found in rs-AMB isolate Kw1661/19 with premature termination of the ERG2 protein (Table 2). High levels of ergosterol were present among reference C. kefyr ATCC 28838 and 4 selected isolates WT for AMB by Etest (Table 2).

Compared to the WT sequence of ERG6 (C-24 sterol methyltransferase) protein from C. kefyr ATCC 26548 (GenBank accession number AP012215), one or more nonsynonymous mutations were found among 11 of 81 isolates. These included an F363L mutation in 3 isolates (1 WT for AMB and 2 rs-AMB), K163Q in 2 isolates WT for AMB, D168G in 1 isolate WT for AMB, D4E plus K163Q mutations in 4 isolates WT for AMB, and D4E plus K163R mutations in 1 isolate WT for AMB by Etest. However, 3 selected isolates (Kw197/13, Kw3153/14, and Kw3267/17) WT for AMB by Etest but containing D168G or D4E plus K163Q or K163Q mutations contained ergosterol levels nearly similar to the reference C. kefyr ATCC 28838 and another isolate (Kw1672/11) WT for AMB by Etest and ERG6 protein sequence (Table 2). A few synonymous mutations, including heterozygosity, were also detected in *ERG6* among some isolates.

Since rs-AMB isolate Kw2153/18 with WT ERG2 protein (a role for the F363L mutation in ERG6 in rs-AMB was ruled out, as this mutation was also present in 1 isolate WT for AMB) completely lacked ergosterol and since ERG3 and ERG11 genes could also be involved in conferring rs-AMB in *Candida* spp. (37–40), these two gene sequences were also analyzed from all 8 rs-AMB and 4 selected isolates WT for AMB by Etest. Compared to the WT sequence of ERG3 (sterol Δ^5,6^ desaturase) protein from C. kefyr ATCC 26548 (GenBank accession number AP012214), two nonsynonymous mutations were detected, N313S in rs-AMB isolate (Kw3352/11) and S218P in rs-AMB isolate Kw2153/18, and both isolates lacked ergosterol (Table 2). Compared to the WT sequence of ERG11 (lanosterol 14α-demethylase) protein from C. kefyr ATCC 26548 (GenBank accession number KF964546), three nonsynonymous mutations were detected, both in rs-AMB isolates. Two mutations (K151R and Y227D) were found in isolate Kw135/15, non-WT for fluconazole and AMB, and a K189R mutation in Kw196-11/20, WT for fluconazole but non-WT for AMB. In order to further elucidate the role of these mutations (K189R and K151R plus Y227D) in reduced susceptibility to fluconazole and AMB, the *ERG11* sequence was determined from an additional 20 (all WT for fluconazole) isolates. The data showed that 5 of 20 isolates also contained the K189R mutation in *ERG11*, while K151R or Y227D or any other nonsynonymous mutation was not detected. A few synonymous mutations, including heterozygosity, were also detected among some isolates in *ERG3* as well as in *ERG11*.

No mutation was detected in hot spot 1 (HS-1) or HS-2 of *FKS1* in any of the 12 C. kefyr isolates, including the isolate non-WT for micafungin, as also reported previously (20). All nonsynonymous mutations in *ERG* genes were double confirmed by extraction of DNA again from C. kefyr isolates and PCR sequencing of the respective loci.

### Sterol compositions in C. kefyr isolates.

Ergosterol comprised 64% to 90% of the total cell sterol in reference strain C. kefyr ATCC 28838 and 4 selected clinical C. kefyr isolates WT for AMB by Etest (Table 3). Interestingly, reference C. kefyr ATCC 28838, Kw197/13, and Kw3267/17 contained lower (64.0% to 72.8%) ergosterol levels and 12.4% to 16.1% ergosta-5,7-dienol levels, and all these three strains contained a nonsynonymous mutation (A113S) in *ERG2* (Table 3). Of 8 rs-AMB isolates, only isolate Kw1661/19 contained ergosterol levels similar to isolates WT for AMB, while the remaining 7 isolates completely lacked ergosterol (Table 3). Of the latter 7 isolates, 6 isolates accumulated ergosta-type sterols [ergosta-8-enol; ergosta-8,22-dienol; ergosta-5,8,22-trienol; ergosta-8,24(28)-dienol or fecosterol and ergosta-5,8,24(28)-trienol], indicating loss of ERG2 protein function, and all these 6 isolates contained nonsynonymous mutations in *ERG2* (Tables 2 and 3). Furthermore, the remaining rs-AMB isolate (Kw2153/18) lacking ergosterol accumulated ergosta-7,22-dienol, episterol [ergosta-7,24(28)-dienol] and ergosta-8,22-dienol, indicating loss of ERG3 protein function. Indeed, this Kw2153/18 isolate contained a novel S218P nonsynonymous mutation in *ERG3* which was absent in other rs-AMB isolates or other isolates WT for AMB by Etest. Although another isolate, Kw3352/11, also contained another N313S nonsynonymous mutation in *ERG3*, sterol profiles did not indicate loss of ERG3 protein function in this isolate. Interestingly, none of the isolates accumulated lanosterol, including Kw135/15 (with K151R plus Y227D mutations in *ERG11*), non-WT for fluconazole (Table 3).

**TABLE 3:** Total C. kefyr cell sterol composition in 4 isolates WT for amphotericin B and 8 isolates with rs-AMB

Type of sterol detected	% of different sterols present in total cell sterols^*a*^ of C. kefyr isolate no.:												
	ATCC 28838	Kw1672/11	Kw197/13	Kw3153/14	Kw3267/17	Kw3352/11	Kw135/15	Kw2327/17	Kw1075/18	Kw2153/18	Kw1661/19	Kw196-11/20	Kw20-12/20
Ergosta-5,8,22,24(28)-tetraenol	1.0 ± 0.5	2.2 ± 0.7	1.4 ± 0.5	2.4 ± 1.6	0.3 ± 0.2	2.1 ± 0.6	2.0 ± 0.5	1.9 ± 0.4	2.1 ± 0.8		1.4 ± 0.4	0.9 ± 0.6	1.0 ± 0.3
Ergosta-5,8,22-trienol	0.4 ± 0.3	0.5 ± 0.1	0.7 ± 0.2	1.1 ± 0.5	0.2 ± 0.1	**33.4 **±** 4.4**	**28.9 **±** 4.1**	**24.4 **±** 2.6**	**41.4 **±** 6.0**		0.7 ± 0.3	**37.0 **±** 1.5**	**35.4 **±** 3.1**
Ergosta-8,22-dienol						**25.9 **±** 4.6**	**21.8 **±** 2.8**	**21.1 **±** 2.3**	**24.6 **±** 3.1**	**12.0 **±** 1.1**		**20.7 **±** 0.5**	**19.7 **±** 3.6**
Zymosterol (Cholesta-8,24-dienol)	1.3 ± 0.8	2.2 ± 0.2	**8.5 **±** 2.6**	**5.6 **±** 2.8**	2.8 ± 1.7						4.5 ± 1.6		
Ergosterol	**70.5 **±** 7.4**	**85.2 **±** 0.9**	**64.0 **±** 2.3**	**85.9 **±** 2.3**	**72.8 **±** 5.7**						**77.9 **±** 0.3**		
Ergosta-5,8,24(28)-trienol						**10.3 **±** 2.6**	**9.6 **±** 2.2**	**8.4 **±** 0.9**	**12.6 **±** 1.0**			**10.2 **±** 2.2**	**8.8 **±** 1.9**
Ergosta-8,22,24(28)-trienol										1.7 ± 0.4			
Ergosta-5,7,22,24(28)-tetraenol	1.5 ± 0.5	2.3 ± 0.2	0.8 ± 0.6		0.8 ± 0.5						0.5 ± 0.3		
Ergosta-7,22-dienol										**67.9 **±** 2.3**			
Ergosta-5,8-dienol						0.4 ± 0.3	0.9 ± 0.4	1.4 ± 0.3	0.5 ± 0.3				1.0 ± 0.4
Fecosterol [Ergosta-8,24(28)-dienol]	4.5 ± 2.4	1.3 ± 0.6	3.8 ± 0.4	1.1 ± 0.3	2.0 ± 1.0	**9.1 **±** 2.7**	**14.6 **±** 3.3**	**26.4 **±** 1.9**	**5.5 **±** 0.4**	1.8 ± 1.0	1.0 ± 0.5	**10.8 **±** 5.2**	**9.8 **±** 2.9**
Ergosta-5,7,24(28)-trienol	1.2 ± 0.4	1.0 ± 0.1	1.3 ± 0.7		2.0 ± 1.3						1.2 ± 0.5		
Ergosta-8-enol						**14.9 **±** 4.1**	**20.0 **±** 3.7**	**12.7 **±** 3.9**	**11.7 **±** 1.0**	0.8 ± 0.3		**19.4 **±** 6.5**	**22.5 **±** 0.7**
Ergosta-5,7-dienol	**12.4 **±** 4.4**	2.3 ± 0.9	**14.3 **±** 1.3**	0.3 ± 0.0	**16.1 **±** 3.2**						**10.4 **±** 1.0**		
Episterol [Ergosta-7,24(28)-dienol]	2.3 ± 0.5	0.4 ± 0.2	3.6 ± 1.1	1.0 ± 0.6	1.6 ± 0.8					**13.4 **±** 1.4**	1.3 ± 0.4		
Ergosta-7-enol										1.7 ± 0.8			
Lanosterol	4.0 ± 0.7	2.2 ± 1.2	0.5 ± 0.3	1.0 ± 0.7	0.8 ± 0.4	1.3 ± 0.7	1.6 ± 0.2	0.3 ± 0.1	0.1 ± 0.0	0.4 ± 0.2	0.7 ± 0.3	0.3 ± 0.1	0.5 ± 0.2
4,4-Dimethyl cholesta-8,24-dienol	0.8 ± 0.9	0.3 ± 0.3	0.9 ± 0.5	1.2 ± 0.9	0.3 ± 0.0	0.4 ± 0.3	0.3 ± 0.1	0.8 ± 0.2	0.4 ± 0.2	0.4 ± 0.1	0.3 ± 0.1	0.1 ± 0.1	0.2 ± 0.1
Unknown sterol						2.3 ± 1.4	0.3 ± 0.2	2.7 ± 1.1	0.2 ± 0.2			0.8 ± 1.2	1.0 ± 0.5
Eburicol	0.2 ± 0.2	0.2 ± 0.2	0.2 ± 0.1	0.4 ± 0.4	0.4 ± 0.3	0.4 ± 0.3	0.2 ± 0.2	0.1 ± 0.1	0.8 ± 0.6		0.2 ± 0.2		

a C. kefyr ATCC 28838 was used as a reference strain for determining total cell sterol composition among clinical C. kefyr isolates. Mean percentage sterol values are from 3 replicates (with standard deviations). Values of >5% total cell sterol are shown in bold.

## DISCUSSION

The high mortality rates associated with invasive *Candida* and other yeast infections are mainly due to availability of a limited number of effective antifungal drugs and emergence of drug-resistant and multidrug-resistant *Candida* and other yeast species (2, 4, 6, 9, 41). Although resistance to AMB is rarely observed in clinical C. albicans isolates or other frequently encountered *Candida* species (such as C. parapsilosis and C. tropicalis), it is increasingly being reported in rare yeast species (such as C. lusitaniae, *C. haemulonii*, and C. kefyr) and is a matter of major concern with C. glabrata and multidrug-resistant C. auris for effective clinical treatment (11, 16, 39, 40, 42–46). Previous studies carried out on clinical rs-AMB isolates and *in vitro*-generated strains have identified *ERG1*, *ERG2*, *ERG3*, *ERG6*, and/or *ERG11* encoded proteins involved in ergosterol biosynthesis as major targets conferring rs-AMB in some *Candida* spp. (11, 16, 37, 38, 40, 47–49).

In this study, AFST of 81 C. kefyr isolates by Etest identified 8 rs-AMB and 73 isolates WT for AMB. However, AFST results by SYO were concordant for only 10 of 12 selected C. kefyr isolates. Although SYO correctly identified 8 of 8 rs-AMB isolates, 2 of 4 isolates WT for AMB by Etest scored as rs-AMB (categorical agreement for 10 of 12 or 83.3% of the isolates). Both isolates (Kw197/13 and Kw3267/17) with discordant results contained ergosterol as their major cell sterol, indicating susceptibility to AMB. Concordance between different AFST methods for AMB among yeast species is not perfect, especially the commercial systems (50–52). Previous studies have also shown discordant AFST results for C. albicans isolates with defects in *ERG3* (40, 53). The essential agreement (±2 2-fold dilutions) between Etest and SYO was even poorer (7 of 12, or 58.3%). In a recent study on C. auris isolates, an essential agreement (±1 dilution) of only 29% and a categorical agreement of 11% were obtained between SYO and the reference methodology by the Clinical and Laboratory Standards Institute for AMB (54). Only use of a higher wild-type upper limit for the MIC or 50% or 75% growth inhibition during SYO testing improved categorical agreement to ≥97% (54).

The identical *ERG2*-encoded protein sequence from 4 C. kefyr strains (ATCC 26548, 100656-19, DMKU3-1042, and FIM1) were used as reference in this study. A total of 70 C. kefyr isolates from Kuwait that were WT for AMB by Etest also contained the wild-type sequence for the *ERG2*-encoded protein, which further supports the use of the C. kefyr ATCC 26548 sequence as reference. The results of *ERG2* sequencing and sterol analyses were remarkable for 7 of 8 rs-AMB isolates. Six rs-AMB isolates contained a nonsynonymous mutation (G90C, M93I, E105K, L107S, G121C, or H155R), completely lacked ergosterol, and accumulated ergosta-8-enol, ergosta-8,22-dienol, ergosta-5,8,22-trienol, and ergosta-8,24(28)-dienol (fecosterol), clearly showing a block of ERG2 activity in the ergosterol biosynthetic pathway (46). Nonsynonymous mutations in *ERG2* causing loss of ergosterol and accumulation of ergosta-type sterols have also previously been linked to loss of ERG2 protein function and rs-AMB in C. glabrata (11, 47). Although the above six nonsynonymous mutations were absent in 73 isolates WT for AMB, 3 WT isolates as well as C. kefyr isolate (ATCC 28838) used as reference for sterol analyses in this study contained another (A113S) nonsynonymous mutation in *ERG2*. Although all 4 isolates WT for AMB and C. kefyr ATCC 28838 analyzed for total cell sterols contained ergosterol as the major cell sterol, as expected, 2 of 4 WT isolates with A113S mutation and the reference C. kefyr ATCC 28838 contained slightly lower levels of ergosterol and also accumulated ergosta-5,7-dienol. Interestingly, A113 is also conserved among different yeast species except C. auris (see Fig. S1) which, similar to C. kefyr with the A113S mutation, also accumulated >5% amounts of ergosta-5,7-dienol (16).

One rs-AMB isolate (Kw1661/19) contained a G-to-A transition at nucleotide 616 and deletion of one nucleotide (617T) which caused a frameshift and resulted in premature termination of the *ERG2* transcript at codon 308, while another rs-AMB isolate (Kw2153/18) contained wild-type sequence for ERG2. Although isolate Kw1661/19 contained ergosterol, its levels were similar to WT isolates (ATCC 28838, Kw197/13, and Kw3267/17) containing the A113S nonsynonymous mutation in ERG2, and it also accumulated ergosta-5,7-dienol, indicating that the truncated C-terminal end in ERG2 in this isolate was perhaps causing the same effects as the A113S mutation in some other isolates. It is not clear at present how the A113S mutation or truncation of the C-terminal end in *ERG2* could result in accumulation of ergosta-5,7-dienol, which usually occurs in isolates with reduced activity of the *ERG5*-encoded protein. *ERG5* was not sequenced in this study. Interestingly, nonsynonymous mutations G90C, M93I, E105K, L107S, and G121C map within the highly conserved region of ERG2 protein, comprising codons 84 to 132 (C. kefyr codon numbering) among different yeast species (Fig. S1). This extended region includes G118, T120, and G121 (G119, T121, and G122 in C. glabrata codon numbering) that are mutated in rs-AMB C. glabrata isolates (11, 47).

Although several nonsynonymous mutations were detected in *ERG6* either singly or in combination, they were found in some rs-AMB as well as in some isolates WT for AMB, and the sterol profiles of the 12 selected isolates, including isolates WT for AMB with an *ERG6* mutation(s), did not show loss of ERG6 protein function, as they did not accumulate cholesta-type sterols. This is contrary to the data obtained with rs-AMB C. glabrata and C. auris. The rs-AMB in C. glabrata is mainly associated with loss of ERG6 protein function (11, 48, 55). Loss of ERG6 protein not only confers rs-AMB but also affects cell wall integrity and calcineurin signaling in C. glabrata (56). The only known molecular mechanism of rs-AMB in C. auris confirmed so far also involves loss of ERG6 protein function (16, 57).

Sterol analyses of the 12 selected C. kefyr isolates indicated loss of ERG3 protein function in rs-AMB isolate Kw2153/18, as it accumulated ergosta-7,22-dienol, ergosta-8,22-dienol, and episterol. Indeed, PCR sequencing of *ERG3* from 12 selected isolates identified a novel (S218P) nonsynonymous mutation in isolate Kw2153/18, which was absent in the remaining 12 isolates. Although isolate Kw3352/11 also contained another (N313S) nonsynonymous mutation in *ERG3*, it likely represented a mere polymorphism, as this isolate contained C_5_ desaturated sterols, indicating that the ERG3 protein is active. Furthermore, the role of N313S mutation in ERG3 in rs-AMB is also unlikely, as isolate Kw3352/11 contained a G121C mutation in *ERG2* and the sterol profiles indicated loss of ERG2 rather than ERG3 protein function (Tables 2 and 3). The G121 (G122 in C. glabrata) is also mutated (Fig. S1) in one rs-AMB C. glabrata isolate described previously (11). The results of *ERG11* sequencing were unremarkable except for one isolate (Kw135/15) non-WT for fluconazole. Although K151 is conserved across several *Candida* spp. and Y227 is also located within another highly conserved region of *ERG11* (Fig. S2), the effects of K151R and Y227D nonsynonymous mutations in isolate Kw135/15 on *ERG11* activity remain unclear due to lack of lanosterol accumulation. The nonsynonymous mutation K189R detected in *ERG11* in rs-AMB isolate Kw196-11/20 is a polymorphism not connected with antifungal resistance, as this alteration was also detected in an additional 5 of 20 C. kefyr isolates WT for all four antifungal drugs. Furthermore, R189 is also found in *ERG11* from C. tropicalis and C. auris (Fig. S2). Taken together, the molecular basis of reduced susceptibility to fluconazole in isolates Kw135/15 and Kw2153/18 remains unclear.

The molecular basis of rs-AMB in isolate Kw1661/19 remains unknown. It has recently been suggested that, similar to molecular genetic analyses of *ERG* genes, analysis of total cell sterols among clinical yeast isolates can also be used to predict reduced susceptibility to triazoles and polyene antifungal drugs (58). Although isolate Kw1661/19 was scored as rs-AMB by Etest and SYO, our data showed that both of the above (*ERG* genes and sterol analyses) approaches are imperfect. Isolate Kw1661/18 contained a deletion frameshift mutation in *ERG2*, which is indicative of loss of function and hence rs-AMB, but the sterol profiles did not show loss of ERG2 protein function. On the contrary, presence of ergosterol as the major (77.9%) constituent of total cell sterol is indicative of the WT pattern for AMB susceptibility even though *in vitro* AFST results indicated rs-AMB. It is probable that rs-AMB in isolate Kw1661/19 is due to involvement of ergosterol-independent (such as efflux pump) resistance mechanisms.

Our study has a few limitations. (i) The definitive role of *ERG2* mutations in conferring rs-AMB in C. kefyr was not confirmed by gene replacement studies. (ii) *ERG3*, *ERG11* gene sequencing, and sterol analyses were not performed on all C. kefyr isolates. (iii) Other *ERG* genes, such as *ERG4* and *ERG5*, were not analyzed. (iv) Information on treatment of patients yielding C. kefyr isolates with antifungal drugs was not available.

In conclusion, 8 of 74 (10.8%) patients yielded rs-AMB C. kefyr isolates in Kuwait. Six rs-AMB isolates contained nonsynonymous mutations in *ERG2*, and their total cell sterol contents were consistent with loss of *ERG2*-encoded protein function. These specific mutations were also absent in 73 isolates WT for AMB. One rs-AMB isolate contained a nonsynonymous mutation in *ERG*3 with concomitant total cell sterol profiles, while the molecular basis of rs-AMB in another isolate remained unknown even though it contained a frameshift mutation in *ERG2* near the C-terminal end; this change was not associated with corresponding changes in total cell sterol profiles. Our data showed that *ERG2* is a major target conferring rs-AMB in clinical C. kefyr isolates in Kuwait.

## MATERIALS AND METHODS

### Clinical C. kefyr isolates, culture conditions, and identification.

A total of 81 C. kefyr isolates recovered from various clinical specimens (Table S1) obtained from 74 patients admitted in different hospitals across Kuwait and obtained during 2011 to 2020 were used. C. kefyr ATCC 28838 and C. kefyr ATCC 26548 (CBS 6556), susceptible to fluconazole, voriconazole, micafungin, and amphotericin B (20, 36), were used as reference strains. The clinical specimens yielding C. kefyr were collected from adult patients in different hospitals after obtaining informed verbal consent only as part of routine patient care and diagnostic workup for cultivation, identification, and antifungal drug susceptibility testing (AFST) of fungal pathogens.

The specimens were cultured in Bactec Plus blood culture bottles (Beckton Dickinson, Sparks, MD, USA) and/or Sabouraud dextrose agar (Difco) plates supplemented with chloramphenicol (50 μg/mL) as described previously (20, 59). All isolates were identified to the species level by using the Vitek 2 yeast identification system (bioMérieux, Marcy-l’Etoile, France). DNA from all isolates was extracted by using a commercial kit (Gentra Puregene yeast DNA extraction kit, Qiagen, Hilden, Germany) or by the rapid boiling method using Chelex-100, as described previously (60). Molecular identity of each isolate was established by PCR amplification of internal transcribed spacer (ITS) region of rDNA by using C. kefyr-specific CKEF (5′-GCTCGTCTCTCCAGTGGACATA-3′) and CKER (5′-ACTCACTACCAAACCCAAAGGT-3′) primers, as described previously (20). PCR sequencing of rDNA was also done for 30 selected C. kefyr isolates, including all drug-resistant isolates, by using pan-fungal primers, as described previously (61).

### Antifungal drug susceptibility testing.

The AFST was initially performed for all C. kefyr isolates against AMB, fluconazole, voriconazole, and micafungin by Etest (bioMérieux SA, Marcy-l’-Etoile, France), following the manufacturer's instructions. Reference strains of C. parapsilosis (ATCC 22019) and C. albicans (ATCC 90028) were also used for quality control. Since there are no susceptibility breakpoints available for C. kefyr, epidemiological cutoff values were used for interpreting the MIC values for AMB, fluconazole, voriconazole, and micafungin. Isolates with MICs of ≤1.0 μg/mL, ≤1.0 μg/mL, ≤0.03 μg/mL, and ≤0.5 μg/mL were considered WT, while isolates with MICs of >1.0 μg/mL, >1.0 μg/mL, >0.03 μg/mL, and >0.5 μg/mL were considered non-WT for AMB, fluconazole, voriconazole, and micafungin, respectively (34, 35). All 8 rs-AMB isolates and 4 selected isolates wild-type for AMB were also tested by using the SYO broth dilution colorimetric method by following the manufacturer's instructions, as described previously (35).

### Sequencing of *ERG* genes participating in ergosterol synthesis.

The complete coding sequences of *ERG2* and *ERG6* genes, frequently implicated in rs-AMB among *Candida* spp. (11, 16, 47, 48), were obtained for all C. kefyr isolates by PCR amplification from genomic DNA, followed by bidirectional amplicon sequencing by using gene-specific primers. The *ERG2* gene was amplified by using CkefERG2F (5′-GGATTTAGGGGAATTAAAGTAG-3′) and CkefERG2R (5′-CTACCGTCAGTACACAAGTGTA-3′) primers and amplification reaction and cycling conditions described previously (62). A PCR product purification kit (Qiagen, Hilden, Germany) was used according to kit instructions to purify the amplicons, and both strands were sequenced by using forward (CkefERG2F or CkefERG2FS, 5′-GAATTAAAGTAGTTCTCTACTCT-3′) or reverse (CkefERG2RS1, 5′-ACCGTCAGTACACAAGTGTATAT-3′ or CkefERG2RS2, 5’AACTTCAGGAACTAGATTGTGT-3′) primers together with a BigDye Terminator v3.1 cycle sequencing kit (Applied Biosystems, Austin, TX, USA) and an ABI 3130*xl* Genetic Analyzer according to the manufacturer’s instructions (Applied Biosystems), as described previously (11, 20). The complete *ERG2* sequences of ~846 bp were assembled and compared with the corresponding sequences from reference strain C. kefyr ATCC 26548 by using the Clustal omega program (https://www.ebi.ac.uk/Tools/msa/clustalo/).

The *ERG6* coding and the flanking noncoding regions were amplified and sequenced as two overlapping fragments. The N-terminal fragment was amplified by using CkefERG6F1 (5′-AATTTGCGCAGTAAGAGAGAGA-3′) and CkefERG6R1 (5′-GACTGGGTACATCTTTGGAATA-3′) primers, while the C-terminal fragment was amplified by using CkefERG6F2 (5′-GAAATTGAACTAGGTGACGGTA-3′) and CkefERG6R2 (5′-GTTTGAGACCTTCCGCTCCACT-3′) primers and PCR amplification reaction and cycling conditions described previously (62). The amplicons were purified as described above, and both strands were sequenced by using the gene-specific primers and the sequencing protocol described previously (11, 20). The sequencing primers for the N-terminal fragment included forward (CkefERG6FS1, 5′-TGCGCAGTAAGAGAGAGACCA-3′) and reverse (CkefERG6RS1, 5′-GACTGGGTACATCTTTGGAATA-3′ or CkefERG6RS2, 5′-AGCACAAGGTCGTTCTCCTTGA-3′) primers. The C-terminal fragment was sequenced by using forward (CkefERG6F2) and reverse (CkefERG6RS3, 5′-ACCTTCCGCTCCACTTTTTTCT-3′) primers. The complete *ERG6* coding sequences of 1,128 bp were assembled and compared with the corresponding sequences from reference C. kefyr ATCC 26548 by using the Clustal omega program.

The *ERG3* and *ERG11* genes were also amplified and sequenced from 12 selected isolates, including all rs-AMB isolates. The *ERG3* gene was amplified and sequenced as two overlapping fragments. The N-terminal fragment was amplified by using CkefERG3F1 (5′-GAAAGAGTGTGTTCTAGCTGA-3′) and CkefERG3R1 (5′-CAATGGGAATAGCATTGGGTA-3′) primers, while the C-terminal fragment was amplified by using CkefERG3F2 (5′-CAGTCGATGGTTTCATGCAA-3′) and CkefERG3R2 (5′-TTTACATTGAGACCATCGATAT-3′) primers; the amplicons were purified and both strands were sequenced by using the gene-specific primers as described above for ERG2 and ERG6. The sequencing primers for the N-terminal fragment included forward (CkefERG3F or CkefERG3FS1, 5′-CATATTCGGTTGGTTGTTGTA-3′) and reverse (CkefERG3R1 or CkefERG3RS1, 5′-ATGGTTAAACACTGCCTTGTCA-3′) primers. The C-terminal fragment was sequenced with the same amplification primers (CkefERG3F2 or CkefERG3R2). The complete *ERG3* coding sequences of 1,059 bp were assembled and were compared with the corresponding sequences from reference C. kefyr ATCC 26548 by using the Clustal omega program. The complete *ERG11* coding sequence of 1,581 bp was obtained as described previously (20).

### Total cell sterol analyses.

The total cell sterol content of 12 selected C. kefyr isolates was determined as described previously (11, 49). Briefly, the yeast cells were grown for 16 h in morpholinepropanesulfonic acid-buffered RPMI medium (pH 7.0) containing 2% glucose. Cells were harvested and nonsaponifiable lipids were extracted, dried in a vacuum centrifuge, and were derivatized with trimethylsilane (TMS). The TMS-derivatized sterols were analyzed by gas chromatography-mass spectrometry (GC-MS). The GC-MS data files were analyzed, and results of three replicates from each sample were used to calculate the mean percentage ± standard deviation for each sterol (11, 49).

### PCR sequencing of hot spot-1 and hot spot-2 of *FKS1*.

The hot spot-1 and hot spot-2 regions of the *FKS1* gene, commonly mutated in echinocandin-resistant *Candida* spp. and other yeast species (63), were also amplified and sequenced from all 12 C. kefyr isolates by using specific amplification and sequencing primers, as described previously (20).

### Ethics statement.

The clinical specimens yielding C. kefyr isolates analyzed in this study were obtained from hospitalized patients admitted in different hospitals across Kuwait after obtaining informed verbal consent only for culture and antifungal susceptibility testing of fungal pathogens as part of routine diagnostic workup. The isolates were used in the Department of Microbiology, Faculty of Medicine, Kuwait University, for identification of fungal pathogens and their susceptibility to antifungal drugs. The results from deidentified samples are described in this paper without revealing patient identity. The study and the consent procedure were approved by the Health Sciences Center Ethics Committee, Kuwait University (approval number VDR/EC/3691).

### Data availability.

The DNA sequence data reported in this study have been submitted to GenBank under accession numbers OQ520304 to OQ520311 and OQ542694 to OQ542744.
