# Supplementary material for: Molecular Characterization and Sterol Profiles Identify Nonsynonymous Mutations in ERG2 as a Major Mechanism Conferring Reduced Susceptibility to Amphotericin B in Candida kefyr
Source: Microbiol Spectr. 2023 Jun 26;11(4):e01474-23. doi: 10.1128/spectrum.01474-23 (PMC10434000; doi:10.1128/spectrum.01474-23)
Supplement: Supplemental file 1 — Table S1 and Fig. S1 and S2. Download spectrum.01474-23-s0001.docx, DOCX file, 0.03 MB [file spectrum.01474-23-s0001.docx]

**TABLE S1.** No. of *C. kefyr* isolates obtained from different clinical specimens of patients used in this study

| Specimen | No. of *C. kefyr* | No. of |
| --- | --- | --- |
| type | isolates | patients |
| Urine | 40 | 36 |
| Sputum | 16 | 15 |
| Abdominal/peritoneal fluid | 6 | 4 |
| Blood | 4 | 4 |
| Bronchoalveolar lavage | 3 | 3 |
| Endotracheal secretion | 3 | 3 |
| Tracheal aspirate | 2 | 2 |
| Vaginal swab | 2 | 2 |
| Ear swab | 1 | 1 |
| Gastric aspirate | 1 | 1 |
| Oral cavity swab | 1 | 1 |
| Tissue biopsy | 1 | 1 |
| Unknown | 1 | 1 |
| **Total** | **81** | **74** |

**FIG. S1**. Sequence alignment of reference and clinical isolates of *Candida kefyr, Saccharomyces cerevisiae, Candida glabrata, Candida auris, Candida albicans* and *Candida dubliniensis* C-8 sterol isomerase (*ERG2*) proteins. Positions of amino acid substitutions identified in clinical *C. kefyr* and *C. glabrata* isolates connected with reduced susceptibility to amphotericin B are highlighted in red. The position of A113S substitution in *C. kefyr* and the corresponding change in *C. auris* are highlighted in green. The frame shift and premature termination in *C. kefyr* 1661/19 at codons 206-208 is underlined. GenBank accession numbers for reference sequences are as follows: *C. kefyr* ATCC26548, AP012218; *S. cerevisiae* S288C, NP013929; *C. glabrata* CBS138, XP449233; *C. glabrata* Kw844/10, SPQ27713; *C. glabrata* Kw3060/15, SPQ27719; *C. auris* CBS10913, UVT84599; *C. albicans*, XP718988 and *C. dubliniensis*, CAX44336. Sequence data for *C. glabrata* isolates CG852 and CG872 are only available from Reference 44. The clustal consensus sequence indicates conserved residues (*).

C.kefyr ATCC26548 MK-LLKLGLFFAAAYYVMNSLLYTWLPRNYIFDPKDLNQIVNTALAEYDLESESFDLKVM 59

C.kefyr ATCC28838 MK-LLKLGLFFAAAYYVMNSLLYTWLPRNYIFDPKDLNQIVNTALAEYDLESESFDLKVM 59

C.kefyr Kw1661/19 MK-LLKLGLFFAAAYYVMNSLLYTWLPRNYIFDPKDLNQIVNTALAEYDLESESFDLKVM 59

C.kefyr Kw197/13 MK-LLKLGLFFAAAYYVMNSLLYTWLPRNYIFDPKDLNQIVNTALAEYDLESESFDLKVM 59

C.kefyr Kw3267/17 MK-LLKLGLFFAAAYYVMNSLLYTWLPRNYIFDPKDLNQIVNTALAEYDLESESFDLKVM 59

C.kefyr Kw20-12/20 MK-LLKLGLFFAAAYYVMNSLLYTWLPRNYIFDPKDLNQIVNTALAEYDLESESFDLKVM 59

C.kefyr Kw196-1/20 MK-LLKLGLFFAAAYYVMNSLLYTWLPRNYIFDPKDLNQIVNTALAEYDLESESFDLKVM 59

C.kefyr Kw1075/18 MK-LLKLGLFFAAAYYVMNSLLYTWLPRNYIFDPKDLNQIVNTALAEYDLESESFDLKVM 59

C.kefyr Kw2327/17 MK-LLKLGLFFAAAYYVMNSLLYTWLPRNYIFDPKDLNQIVNTALAEYDLESESFDLKVM 59

C.kefyr Kw135/15 MK-LLKLGLFFAAAYYVMNSLLYTWLPRNYIFDPKDLNQIVNTALAEYDLESESFDLKVM 59

C.kefyr Kw3352/11 MK-LLKLGLFFAAAYYVMNSLLYTWLPRNYIFDPKDLNQIVNTALAEYDLESESFDLKVM 59

C.kefyr Kw1672/11 MK-LLKLGLFFAAAYYVMNSLLYTWLPRNYIFDPKDLNQIVNTALAEYDLESESFDLKVM 59

C.kefyr Kw3153/14 MK-LLKLGLFFAAAYYVMNSLLYTWLPRNYIFDPKDLNQIVNTALAEYDLESESFDLKVM 59

C.kefyr Kw2153/18 MK-LLKLGLFFAAAYYVMNSLLYTWLPRNYIFDPKDLNQIVNTALAEYDLESESFDLKVM 59

S.cerevisiae S288C MK-FFPLLLLIGVVGYIMNVLFTTWLPTNYMFDPKTLNEICNSVISKHNAA-EGLSTEDL 58

C.glabrata CG852 MKFFINLLLLVAGVGYLLNSLYDSWLPRNYIFDPKTLNEICNGVLAKHNGSDASASTESF 60

C.glabrata CBS138 MKFFINLLLLVAGVGYLLNSLYDSWLPRNYIFDPKTLNEICNGVLAKHNGSDASASTESL 60

C.glabrata Kw844/10 MKFFINLLLLVAGVGYLLNSLYDSWLPRNYIFDPKTLNEICNGVLAKHNGSDASASTESL 60

C.glabrata Kw3060/15 MKFFINLLLLVAGVGYLLNSLYDSWLPRNYIFDPKTLNEICNGVLAKHNGSDASASTESL 60

C.glabrata CG872 MKFFINLLLLVAGVGYLLNSLYDSWLPRNYIFDPKTLNEICNGVLAKHNGSDASASTESL 60

C.auris CBS10913 MNWWLKIIAIFVAVYAVLEASYQTWLPSHYVFDKQVLQELVQQTLKENPGADAK----TL 56

C.albicans SC5314 MK-LLLVGIIPIALYAIFNYLFYTWLPTNYLFDKQVLQELVQETLKDHLDGNAT----AI 55

C.dubliniensis CD36 MK-LLLIGIIPIALYAIFNYLFYTWLPTNYLFDKEILQQLVQETLKDHSDGNST----AI 55

* *** * ** *

C.kefyr ATCC26548 LQDIRDRLANHYGEEYINKYVEEEWVFNNAGGAMGQMIILHASISEYLIFFGSAVGTEGH 119

C.kefyr ATCC28838 LQDIRDRLANHYGEEYINKYVEEEWVFNNAGGAMGQMIILHASISEYLIFFGSSVGTEGH 119

C.kefyr Kw1661/19 LQDIRDRLANHYGEEYINKYVEEEWVFNNAGGAMGQMIILHASISEYLIFFGSAVGTEGH 119

C.kefyr Kw197/13 LQDIRDRLANHYGEEYINKYVEEEWVFNNAGGAMGQMIILHASISEYLIFFGSSVGTEGH 119

C.kefyr Kw3267/17 LQDIRDRLANHYGEEYINKYVEEEWVFNNAGGAMGQMIILHASISEYLIFFGSSVGTEGH 119

C.kefyr Kw20-12/20 LQDIRDRLANHYGEEYINKYVEEEWVFNNA**C**GAMGQMIILHASISEYLIFFGSAVGTEGH 119

C.kefyr Kw196-1/20 LQDIRDRLANHYGEEYINKYVEEEWVFNNAGGAMGQMIILHASISEYSIFFGSAVGTEGH 119

C.kefyr Kw1075/18 LQDIRDRLANHYGEEYINKYVEEEWVFNNAGGAMGQMIILHASISEYLIFFGSAVGTEGH 119

C.kefyr Kw2327/17 LQDIRDRLANHYGEEYINKYVEEEWVFNNAGGA**I**GQMIILHASISEYLIFFGSAVGTEGH 119

C.kefyr Kw135/15 LQDIRDRLANHYGEEYINKYVEEEWVFNNAGGAMGQMIILHASIS**K**YLIFFGSAVGTEGH 119

C.kefyr Kw3352/11 LQDIRDRLANHYGEEYINKYVEEEWVFNNAGGAMGQMIILHASISEYLIFFGSAVGTEGH 119

C.kefyr Kw1672/11 LQDIRDRLANHYGEEYINKYVEEEWVFNNAGGAMGQMIILHASISEYLIFFGSAVGTEGH 119

C.kefyr Kw3153/14 LQDIRDRLANHYGEEYINKYVEEEWVFNNAGGAMGQMIILHASISEYLIFFGSAVGTEGH 119

C.kefyr Kw2153/18 LQDIRDRLANHYGEEYINKYVEEEWVFNNAGGAMGQMIILHASISEYLIFFGSAVGTEGH 119

S.cerevisiae S288C LQDVRDALASHYGDEYINRYVKEEWVFNNAGGAMGQMIILHASVSEYLILFGTAVGTEGH 118

C.glabrata CG852 LIDVRDALAKHYGDEYINEYTRDAWVFNNAGGAMGQMIILHASISEYVILFGTAVGTEGH 120

C.glabrata CBS138 LIDVRDALAKHYGDEYINEYTRDAWVFNNAGGAMGQMIILHASISEYVILFGTAVGTEGH 120

C.glabrata Kw844/10 LIDVRDALAKHYGDEYINEYTRDAWVFNNAGGAMGQMIILHASISEYVILFGTAVGTEGH 120

C.glabrata Kw3060/15 LIDVRDALAKHYGDEYINEYTRDAWVFNNAGGAMGQMIILHASISEYVILFGTAVGTE**S**H 120

C.glabrata CG872 LIDVRDALAKHYGDEYINEYTRDAWVFNNAGGAMGQMIILHASISEYVILFGTAVGTEGH 120

C.auris CBS10913 MVELTPKLQNAYP-GLINDLNFDDWVFNNAGGAMGNMIILHASISEYLIFFGTSVGTEGH 115

C.albicans SC5314 MIDLTPKIQKKYP-KIINDLNFDDWVYNNAGGAMGTMFILHASISEYLIFFGTAIGTEGH 114

C.dubliniensis CD36 MIDLTFKIQKQYP-NIINDLNFNDWVYNNAGGAMGTMFILHASISEYLIFFGTAIGTEGH 114

* ** ** *** ** * * ***** * * * ** *** *

C.kefyr ATCC26548 TGVHFADDYFTILYGEQRASLPHNLVPEVYKPGDTHHLQKGYAKQYSMPTGSFALELAQG 179

C.kefyr ATCC28838 TGVHFADDYFTILYGEQRASLPHNLVPEVYKPGDTHHLQKGYAKQYSMPTGSFALELAQG 179

C.kefyr Kw1661/19 TGVHFADDYFTILYGEQRASLPHNLVPEVYKPGDTHHLQKGYAKQYSMPTGSFALELAQG 179

C.kefyr Kw197/13 TGVHFADDYFTILYGEQRASLPHNLVPEVYKPGDTHHLQKGYAKQYSMPTGSFALELAQG 179

C.kefyr Kw3267/17 TGVHFADDYFTILYGEQRASLPHNLVPEVYKPGDTHHLQKGYAKQYSMPTGSFALELAQG 179

C.kefyr Kw20-12/20 TGVHFADDYFTILYGEQRASLPHNLVPEVYKPGDTHHLQKGYAKQYSMPTGSFALELAQG 179

C.kefyr Kw196-1/20 TGVHFADDYFTILYGEQRASLPHNLVPEVYKPGDTHHLQKGYAKQYSMPTGSFALELAQG 179

C.kefyr Kw1075/18 TGVHFADDYFTILYGEQRASLPHNLVPEVYKPGDT**R**HLQKGYAKQYSMPTGSFALELAQG 179

C.kefyr Kw2327/17 TGVHFADDYFTILYGEQRASLPHNLVPEVYKPGDTHHLQKGYAKQYSMPTGSFALELAQG 179

C.kefyr Kw135/15 TGVHFADDYFTILYGEQRASLPHNLVPEVYKPGDTHHLQKGYAKQYSMPTGSFALELAQG 179

C.kefyr Kw3352/11 T**C**VHFADDYFTILYGEQRASLPHNLVPEVYKPGDTHHLQKGYAKQYSMPTGSFALELAQG 179

C.kefyr Kw1672/11 TGVHFADDYFTILYGEQRASLPHNLVPEVYKPGDTHHLQKGYAKQYSMPTGSFALELAQG 179

C.kefyr Kw3153/14 TGVHFADDYFTILYGEQRASLPHNLVPEVYKPGDTHHLQKGYAKQYSMPTGSFALELAQG 179

C.kefyr Kw2153/18 TGVHFADDYFTILYGEQRASLPHNLVPEVYKPGDTHHLQKGYAKQYSMPTGSFALELAQG 179

S.cerevisiae S288C TGVHFADDYFTILHGTQIAALPYATEAEVYTPGMTHHLKKGYAKQYSMPGGSFALELAQG 178

C.glabrata CG852 **V**GVHFADDYFTILKGVQRAALPWEADPEEYFPGMTHHLQKGYAKQYAMDQNSFALELAQG 180

C.glabrata CBS138 TGVHFADDYFTILKGVQRAALPWEADPEEYFPGMTHHLQKGYAKQYAMDQNSFALELAQG 180

C.glabrata Kw844/10 T**S**VHFADDYFTILKGVQRAALPWEADPEEYFPGMTHHLQKGYAKQYAMDQNSFALELAQG 180

C.glabrata Kw3060/15 TGVHFADDYFTILKGVQRAALPWEADPEEYFPGMTHHLQKGYAKQYAMDQNSFALELAQG 180

C.glabrata CG872 **I**GVHFADDYFTILKGVQRAALPWEADPEEYFPGMTHHLQKGYAKQYAMDQNSFALELAQG 180

C.auris CBS10913 TGVHYADDYFTILYGKELAGLPNSIEPEVYSAGDQHHLERGQVKQYAMPEGSYALELAQG 175

C.albicans SC5314 TGVHFADDYFTILTGEQRAAYPGALIPEVYLPGDQHHLPKGHVKQYAMPGESFALELAQG 174

C.dubliniensis CD36 TGVHFADDYFTILTGEQRAAYSGSLIPEIYLPGDQHHLPKGHVKQYSMPGESFALELAQG 174

** ******** * * * * * ** * *** * * *******

C.kefyr ATCC26548 WIPAMLPFGFLDTFSSTLDWYTLIKTVYLTGRDMIKNLVQNAKF 223

C.kefyr ATCC28838 WIPAMLPFGFLDTFSSTLDWYTLIKTVYLTGRDMIKNLVQNAKF 223

C.kefyr Kw1661/19 WIPAMLPFGFLDTFSSTLDWYTLIKT**ST** 207

C.kefyr Kw197/13 WIPAMLPFGFLDTFSSTLDWYTLIKTVYLTGRDMIKNLVQNAKF 223

C.kefyr Kw3267/17 WIPAMLPFGFLDTFSSTLDWYTLIKTVYLTGRDMIKNLVQNAKF 223

C.kefyr Kw20-12/20 WIPAMLPFGFLDTFSSTLDWYTLIKTVYLTGRDMIKNLVQNAKF 223

C.kefyr Kw196-1/20 WIPAMLPFGFLDTFSSTLDWYTLIKTVYLTGRDMIKNLVQNAKF 223

C.kefyr Kw1075/18 WIPAMLPFGFLDTFSSTLDWYTLIKTVYLTGRDMIKNLVQNAKF 223

C.kefyr Kw2327/17 WIPAMLPFGFLDTFSSTLDWYTLIKTVYLTGRDMIKNLVQNAKF 223

C.kefyr Kw135/15 WIPAMLPFGFLDTFSSTLDWYTLIKTVYLTGRDMIKNLVQNAKF 223

C.kefyr Kw3352/11 WIPAMLPFGFLDTFSSTLDWYTLIKTVYLTGRDMIKNLVQNAKF 223

C.kefyr Kw1672/11 WIPAMLPFGFLDTFSSTLDWYTLIKTVYLTGRDMIKNLVQNAKF 223

C.kefyr Kw3153/14 WIPAMLPFGFLDTFSSTLDWYTLIKTVYLTGRDMIKNLVQNAKF 223

C.kefyr Kw2153/18 WIPAMLPFGFLDTFSSTLDWYTLIKTVYLTGRDMIKNLVQNAKF 223

S.cerevisiae S288C WIPCMLPFGFLDTFSSTLDLYTLYRTVYLTARDMGKNLLQNKKF 222

C.glabrata CG852 WIPCMLPFGFLDTFSSTLDLYTLGKTVYLTAKDMIKNLVQNQKF 224

C.glabrata CBS138 WIPCMLPFGFLDTFSSTLDLYTLGKTIYLTAKDMIKNLVQNQKF 224

C.glabrata Kw844/10 WIPCMLPFGFLDTFSSTLDLYTLGKTVYLTAKDMIKNLVQNQKF 224

C.glabrata Kw3060/15 WIPCMLPFGFLDTFSSTLDLYTLGKTVYLTAKDMIKNLVQNQKF 224

C.glabrata CG872 WIPCMLPFGFLDTFSSTLDLYTLGKTVYLTAKDMIKNLVQNQKF 224

C.auris CBS10913 WIPAMLPFGFVSVLTSTLDFHSFGRTVWFTAYDMGKNLLKGKF 218

C.albicans SC5314 WIPAMLPFGFLDTLTSTMDFYTFYLTAYYTGKDMIKNL 212

C.dubliniensis CD36 WIPAMLPFGFLDTLTSTMDFHTFYLTAYFTGKDMIKNLLNGKF 217

*** ****** ** * *

**FIG. S2**. Sequence alignment of reference and clinical isolates of *Candida kefyr, Candida glabrata, Candida auris, Candida parapsilosis, Candida albicans* and *Candida tropicalis* lanosterol 14α-demethylase (*ERG11*) proteins. Positions of novel nonsynonymous amino acid substitutions identified in clinical *C. kefyr* isolate with reduced susceptibility to fluconazole are highlighted in red. The position of K189R polymorphism in *C. kefyr* and the corresponding amino acid in *C. auris* and *C. tropicalis* are highlighted in green. GenBank accession numbers for reference sequences are as follows: *C. kefyr* ATCC26548, KF964546; *C. glabrata* CBS138, L40389; *C. auris* CBS10913, AYN77778; *C. parapsilosis* ATCC22019*,* GQ302972; *C. albicans* SC5314, X13296 and *C. tropicalis* ATCC750, M23673. The clustal consensus sequence indicates conserved residues (*).

C.kefyr ATCC26548 MST-SESFVGKLFEVLQGLLLQFWALTITQRVSIVILLPFVYNIVWQLLYSMRKDRVPLV 59

C.kefyr ATCC28838 MST-SESFVGKLFEVLQGLLLQFWALTITQRVSIVILLPFVYNIVWQLLYSMRKDRVPLV 59

C.kefyr Kw135/15 MST-SESFVGKLFEVLQGLLLQFWALTITQRVSIVILLPFVYNIVWQLLYSMRKDRVPLV 59

C.kefyr Kw1672/11 MST-SESFVGKLFEVLQGLLLQFWALTITQRVSIVILLPFVYNIVWQLLYSMRKDRVPLV 59

C.kefyr Kw3153/14 MST-SESFVGKLFEVLQGLLLQFWALTITQRVSIVILLPFVYNIVWQLLYSMRKDRVPLV 59

C.kefyr Kw2153/18 MST-SESFVGKLFEVLQGLLLQFWALTITQRVSIVILLPFVYNIVWQLLYSMRKDRVPLV 59

C.kefyr Kw20-12/20 MST-SESFVGKLFEVLQGLLLQFWALTITQRVSIVILLPFVYNIVWQLLYSMRKDRVPLV 59

C.kefyr Kw196-11/20 MST-SESFVGKLFEVLQGLLLQFWALTITQRVSIVILLPFVYNIVWQLLYSMRKDRVPLV 59

C.kefyr Kw28-10/12 MST-SESFVGKLFEVLQGLLLQFWALTITQRVSIVILLPFVYNIVWQLLYSMRKDRVPLV 59

C.kefyr Kw823/14 MST-SESFVGKLFEVLQGLLLQFWALTITQRVSIVILLPFVYNIVWQLLYSMRKDRVPLV 59

C.kefyr Kw3619/13 MST-SESFVGKLFEVLQGLLLQFWALTITQRVSIVILLPFVYNIVWQLLYSMRKDRVPLV 59

C.glabrata CBS138 MSTENTSLVVELLEYVKLGLSYFQALPLAQRVSIMVALPFVYTITWQLLYSLRKDRPPLV 60

C.auris CBS10913 MA---------LKDCIVDVVDRFSALPVPVKLAVLILVPIVYNLVWQFVYSLRKDRAPLV 51

C.parapsilosis ATCC22019 MA---------LVDLALHGYNYFMTLSTLQQFGLLVFAPFIYNIIWQLLYSLRKDRVPLV 51

C.albicans SC5314 MA---------IVETVIDGINYFLSLSVTQQISILLGVPFVYNLVWQYLYSLRKDRAPLV 51

C.tropicalis ATCC750 MA---------IVDTAIDGINYFLSLSLTQQITILVVFPFIYNIAWQLLYSLRKDRVPMV 51

* * * * * ** ** **** * *

C.kefyr ATCC26548 FYWIPWVGSAVTYGMRPYEFFEECRQKYGDVFSFVLLGRVMTVYLGPKGHEFVLNARLAD 119

C.kefyr ATCC28838 FYWIPWVGSAVTYGMRPYEFFEECRQKYGDVFSFVLLGRVMTVYLGPKGHEFVLNARLAD 119

C.kefyr Kw135/15 FYWIPWVGSAVTYGMRPYEFFEECRQKYGDVFSFVLLGRVMTVYLGPKGHEFVLNARLAD 119

C.kefyr Kw1672/11 FYWIPWVGSAVTYGMRPYEFFEECRQKYGDVFSFVLLGRVMTVYLGPKGHEFVLNARLAD 119

C.kefyr Kw3153/14 FYWIPWVGSAVTYGMRPYEFFEECRQKYGDVFSFVLLGRVMTVYLGPKGHEFVLNARLAD 119

C.kefyr Kw2153/18 FYWIPWVGSAVTYGMRPYEFFEECRQKYGDVFSFVLLGRVMTVYLGPKGHEFVLNARLAD 119

C.kefyr Kw20-12/20 FYWIPWVGSAVTYGMRPYEFFEECRQKYGDVFSFVLLGRVMTVYLGPKGHEFVLNARLAD 119

C.kefyr Kw196-11/20 FYWIPWVGSAVTYGMRPYEFFEECRQKYGDVFSFVLLGRVMTVYLGPKGHEFVLNARLAD 119

C.kefyr Kw28-10/12 FYWIPWVGSAVTYGMRPYEFFEECRQKYGDVFSFVLLGRVMTVYLGPKGHEFVLNARLAD 119

C.kefyr Kw823/14 FYWIPWVGSAVTYGMRPYEFFEECRQKYGDVFSFVLLGRVMTVYLGPKGHEFVLNARLAD 119

C.kefyr Kw3619/13 FYWIPWVGSAVTYGMRPYEFFEECRQKYGDVFSFVLLGRVMTVYLGPKGHEFVLNARLAD 119

C.glabrata CBS138 FYWIPWVGSAIPYGTKPYEFFEDCQKKYGDIFSFMLLGRIMTVYLGPKGHEFIFNAKLAD 120

C.auris CBS10913 FHWVPWVGSAVVYGMQPYQFFESCREKYGDVFAFVMLGKVMTVYLGPKGHEFVLNAKLAD 111

C.parapsilosis ATCC22019 FYWIPWVGSAVSYGQDPYGFFEQCREKYGDLFSFVMLGRVMTVYLGPKGHEFVFNAKLSD 111

C.albicans SC5314 FYWIPWFGSAASYGQQPYEFFESCRQKYGDVFSFMLLGKIMTVYLGPKGHEFVFNAKLSD 111

C.tropicalis ATCC750 FYWIPWFGSAASYGMQPYEFFEKCRLKYGDVFSFMLLGKVMTVYLGPKGHEFIYNAKLSD 111

* * ** *** ** ** *** * **** * * ** ************ ** * *

C.kefyr ATCC26548 VSAEAAYTHLTTPVFGEGVIYDCSNSRLMDQKKFVKGALTKDAFRKYVPLVTEEVQKYFK 179

C.kefyr ATCC28838 VSAEAAYTHLTTPVFGEGVIYDCSNSRLMDQKKFVKGALTKDAFRKYVPLVTEEVQKYFK 179

C.kefyr Kw135/15 VSAEAAYTHLTTPVFGEGVIYDCSNSRLMDQRKFVKGALTKDAFRKYVPLVTEEVQKYFK 179

C.kefyr Kw1672/11 VSAEAAYTHLTTPVFGEGVIYDCSNSRLMDQKKFVKGALTKDAFRKYVPLVTEEVQKYFK 179

C.kefyr Kw3153/14 VSAEAAYTHLTTPVFGEGVIYDCSNSRLMDQKKFVKGALTKDAFRKYVPLVTEEVQKYFK 179

C.kefyr Kw2153/18 VSAEAAYTHLTTPVFGEGVIYDCSNSRLMDQKKFVKGALTKDAFRKYVPLVTEEVQKYFK 179

C.kefyr Kw20-12/20 VSAEAAYTHLTTPVFGEGVIYDCSNSRLMDQKKFVKGALTKDAFRKYVPLVTEEVQKYFK 179

C.kefyr Kw196-11/20 VSAEAAYTHLTTPVFGEGVIYDCSNSRLMDQKKFVKGALTKDAFRKYVPLVTEEVQKYFK 179

C.kefyr Kw28-10/12 VSAEAAYTHLTTPVFGEGVIYDCSNSRLMDQKKFVKGALTKDAFRKYVPLVTEEVQKYFK 179

C.kefyr Kw823/14 VSAEAAYTHLTTPVFGEGVIYDCSNSRLMDQKKFVKGALTKDAFRKYVPLVTEEVQKYFK 179

C.kefyr Kw3619/13 VSAEAAYTHLTTPVFGEGVIYDCSNSRLMDQKKFVKGALTKDAFRKYVPLVTEEVQKYFK 179

C.glabrata CBS138 VSAEAAYSHLTTPVFGKGVIYDCPNHRLMEQKKFVKGALTKEAFVRYVPLIAEEIYKYFR 180

C.auris CBS10913 VSAEAAYSHLTTPVFGKGVIYDCPNSRLMEQKKFAKTALTKEAFQRYVPRIQEEVLDYFK 171

C.parapsilosis ATCC22019 VSAEDAYQHLTTPVFGKGVIYDCPNARLMEQKKFAKTALTTDSFRRYVPLIRGEILDYFT 171

C.albicans SC5314 VSAEDAYKHLTTPVFGKGVIYDCPNSRLMEQKKFAKFALTTDSFKRYVPKIREEILNYFV 171

C.tropicalis ATCC750 VSAEEAYTHLTTPVFGKGVIYDCPNSRLMEQKKFAKFALTTDSFKTYVPKIREEVLNYFV 171

**** ** ******** ****** * *** * ** * *** * *** * **

C.kefyr ATCC26548 NSANFKIGEKDHGKINVMVTQPEMTIFTASRTLLGKEMREKLDTGFAYLYSDLDKGFTPL 239

C.kefyr ATCC28838 NSANFKIGEKDHGKINVMVTQPEMTIFTASRTLLGKEMREKLDTGFAYLYSDLDKGFTPL 239

C.kefyr Kw135/15 NSANFKIGEKDHGKINVMVTQPEMTIFTASRTLLGKEMREKLDTGFA**D**LYSDLDKGFTPL 239

C.kefyr Kw1672/11 NSANFKIGEKDHGKINVMVTQPEMTIFTASRTLLGKEMREKLDTGFAYLYSDLDKGFTPL 239

C.kefyr Kw3153/14 NSANFKIGEKDHGKINVMVTQPEMTIFTASRTLLGKEMREKLDTGFAYLYSDLDKGFTPL 239

C.kefyr Kw2153/18 NSANFKIGEKDHGKINVMVTQPEMTIFTASRTLLGKEMREKLDTGFAYLYSDLDKGFTPL 239

C.kefyr Kw20-12/20 NSANFKIGEKDHGKINVMVTQPEMTIFTASRTLLGKEMREKLDTGFAYLYSDLDKGFTPL 239

C.kefyr Kw196-11/20 NSANFKIGE**R**DHGKINVMVTQPEMTIFTASRTLLGKEMREKLDTGFAYLYSDLDKGFTPL 239

C.kefyr Kw28-10/12 NSANFKIGE**R**DHGKINVMVTQPEMTIFTASRTLLGKEMREKLDTGFAYLYSDLDKGFTPL 239

C.kefyr Kw823/14 NSANFKIGE**R**DHGKINVMVTQPEMTIFTASRTLLGKEMREKLDTGFAYLYSDLDKGFTPL 239

C.kefyr Kw3619/13 NSANFKIGE**R**DHGKINVMVTQPEMTIFTASRTLLGKEMREKLDTGFAYLYSDLDKGFTPL 239

C.glabrata CBS138 NSKNFKINENNSGIVDVMVSQPEMTIFTASRSLLGKEMRDKLDTDFAYLYSDLDKGFTPI 240

C.auris CBS10913 ACSQFKMNE**R**NNGVANVMKTQPEMTILTASKSLMGDDMRARFDASFAKLYSDLDKGFTPI 231

C.parapsilosis ATCC22019 KSKVFNMKKQKSGVVDVLQSQPEITIFTASRSLLGEAMRKRFDASFAQLYADLDKGFTPI 231

C.albicans SC5314 TDESFKLKEKTHGVANVMKTQPEITIFTASRSLFGDEMRRIFDRSFAQLYSDLDKGFTPI 231

C.tropicalis ATCC750 NDVSFKTKE**R**DHGVASVMKTQPEITIFTASRCLFGDEMRKSFDRSFAQLYADLDKGFTPI 231

* * * *** ** *** * * ** * ** ** ********

C.kefyr ATCC26548 NFVFSHLPLDNYRKRDHAQRTISATYMSLIKERRANNDIQ-DRDLIDTLMKSSTYKDGTK 298

C.kefyr ATCC28838 NFVFSHLPLDNYRKRDHAQRTISATYMSLIKERRANNDIQ-DRDLIDTLMKSSTYKDGTK 298

C.kefyr Kw135/15 NFVFSHLPLDNYRKRDHAQRTISATYMSLIKERRANNDIQ-DRDLIDTLMKSSTYKDGTK 298

C.kefyr Kw1672/11 NFVFSHLPLDNYRKRDHAQRTISATYMSLIKERRANNDIQ-DRDLIDTLMKSSTYKDGTK 298

C.kefyr Kw3153/14 NFVFSHLPLDNYRKRDHAQRTISATYMSLIKERRANNDIQ-DRDLIDTLMKSSTYKDGTK 298

C.kefyr Kw2153/18 NFVFSHLPLDNYRKRDHAQRTISATYMSLIKERRANNDIQ-DRDLIDTLMKSSTYKDGTK 298

C.kefyr Kw20-12/20 NFVFSHLPLDNYRKRDHAQRTISATYMSLIKERRANNDIQ-DRDLIDTLMKSSTYKDGTK 298

C.kefyr Kw196-11/20 NFVFSHLPLDNYRKRDHAQRTISATYMSLIKERRANNDIQ-DRDLIDTLMKSSTYKDGTK 298

C.kefyr Kw28-10/12 NFVFSHLPLDNYRKRDHAQRTISATYMSLIKERRANNDIQ-DRDLIDTLMKSSTYKDGTK 298

C.kefyr Kw823/14 NFVFSHLPLDNYRKRDHAQRTISATYMSLIKERRANNDIQ-DRDLIDTLMKSSTYKDGTK 298

C.kefyr Kw3619/13 NFVFSHLPLDNYRKRDHAQRTISATYMSLIKERRANNDIQ-DRDLIDTLMKSSTYKDGTK 298

C.glabrata CBS138 NFVFPNLPLEHYRKRDHAQQAISGTYMSLIKERREKNDIQ-NRDLIDELMKNSTYKDGTK 299

C.auris CBS10913 NFVFPHLPLPAYWKRDAAQQKISATYMSLINERRKTGDIVPDRDLIDSLMTNSTYKDGVK 291

C.parapsilosis ATCC22019 NFVFPHLPLPHYWKRDAAQQKISETYMTEIARRRETGDIDENRDLIDSLLVNSTYKDGVK 291

C.albicans SC5314 NFVFPNLPLPHYWRRDAAQKKISATYMKEIKSRRERGDIDPNRDLIDSLLIHSTYKDGVK 291

C.tropicalis ATCC750 NFVFPNLPLPHYWRRDAAQRKISAHYMKEIKRRRESGDIDPKRDLIDSLLVNSTYKDGVK 291

**** *** * ** ** ** ** * ** ** ***** * ****** *

C.kefyr ATCC26548 MTDKEIANLLIGVLMGGQHTSAATSAWAILHLAERPDVQQELYEEQMRVLDN---GKKEL 355

C.kefyr ATCC28838 MTDKEIANLLIGVLMGGQHTSAATSAWAILHLAERPDVQQELYEEQMRVLDN---GKKEL 355

C.kefyr Kw135/15 MTDKEIANLLIGVLMGGQHTSAATSAWAILHLAERPDVQQELYEEQMRVLDN---GKKEL 355

C.kefyr Kw1672/11 MTDKEIANLLIGVLMGGQHTSAATSAWAILHLAERPDVQQELYEEQMRVLDN---GKKEL 355

C.kefyr Kw3153/14 MTDKEIANLLIGVLMGGQHTSAATSAWAILHLAERPDVQQELYEEQMRVLDN---GKKEL 355

C.kefyr Kw2153/18 MTDKEIANLLIGVLMGGQHTSAATSAWAILHLAERPDVQQELYEEQMRVLDN---GKKEL 355

C.kefyr Kw20-12/20 MTDKEIANLLIGVLMGGQHTSAATSAWAILHLAERPDVQQELYEEQMRVLDN---GKKEL 355

C.kefyr Kw196-11/20 MTDKEIANLLIGVLMGGQHTSAATSAWAILHLAERPDVQQELYEEQMRVLDN---GKKEL 355

C.kefyr Kw28-10/12 MTDKEIANLLIGVLMGGQHTSAATSAWAILHLAERPDVQQELYEEQMRVLDN---GKKEL 355

C.kefyr Kw823/14 MTDKEIANLLIGVLMGGQHTSAATSAWAILHLAERPDVQQELYEEQMRVLDN---GKKEL 355

C.kefyr Kw3619/13 MTDKEIANLLIGVLMGGQHTSAATSAWAILHLAERPDVQQELYEEQMRVLDN---GKKEL 355

C.glabrata CBS138 MTDQEIANLLIGVLMGGQHTSAATSAWCLLHLAERPDVQEELYQEQMRVLNN---DTKEL 356

C.auris CBS10913 MTDQEVANLLIGVLMGGQHTSASTSAWFLLHLAEQPKLQEELYNEVLSVLAEKGGSLKDL 351

C.parapsilosis ATCC22019 MTDQEIANLLIGVLMGGQHTSATTSAWFLLHLAEKPQLQDELYQEVLNALSGKGGNLDDL 351

C.albicans SC5314 MTDQEIANLLIGILMGGQHTSASTSAWFLLHLGEKPHLQDVIYQEVVELLKEKGGDLNDL 351

C.tropicalis ATCC750 MTDQEIANLLIGVLMGGQHTSASTSAWFLLHLAEQPQLQDDLYEELTNLLKEKGGDLNDL 351

*** * ****** ********* **** *** * * * * * * *

C.kefyr ATCC26548 TYDLLQEMPFLNQTIKETLRLHHPLHSLFRKVMNDMPVPNTSYVVPKGHYVLVSPGYCHL 415

C.kefyr ATCC28838 TYDLLQEMPFLNQTIKETLRLHHPLHSLFRKVMNDMPVPNTSYVVPKGHYVLVSPGYCHL 415

C.kefyr Kw135/15 TYDLLQEMPFLNQTIKETLRLHHPLHSLFRKVMNDMPVPNTSYVVPKGHYVLVSPGYCHL 415

C.kefyr Kw1672/11 TYDLLQEMPFLNQTIKETLRLHHPLHSLFRKVMNDMPVPNTSYVVPKGHYVLVSPGYCHL 415

C.kefyr Kw3153/14 TYDLLQEMPFLNQTIKETLRLHHPLHSLFRKVMNDMPVPNTSYVVPKGHYVLVSPGYCHL 415

C.kefyr Kw2153/18 TYDLLQEMPFLNQTIKETLRLHHPLHSLFRKVMNDMPVPNTSYVVPKGHYVLVSPGYCHL 415

C.kefyr Kw20-12/20 TYDLLQEMPFLNQTIKETLRLHHPLHSLFRKVMNDMPVPNTSYVVPKGHYVLVSPGYCHL 415

C.kefyr Kw196-11/20 TYDLLQEMPFLNQTIKETLRLHHPLHSLFRKVMNDMPVPNTSYVVPKGHYVLVSPGYCHL 415

C.kefyr Kw28-10/12 TYDLLQEMPFLNQTIKETLRLHHPLHSLFRKVMNDMPVPNTSYVVPKGHYVLVSPGYCHL 415

C.kefyr Kw823/14 TYDLLQEMPFLNQTIKETLRLHHPLHSLFRKVMNDMPVPNTSYVVPKGHYVLVSPGYCHL 415

C.kefyr Kw3619/13 TYDLLQEMPFLNQTIKETLRLHHPLHSLFRKVMNDMPVPNTSYVVPKGHYVLVSPGYCHL 415

C.glabrata CBS138 TYDDLQNMPLLNQMIKETLRLHHPLHSLFRKVMRDVAIPNTSYVVPRDYHVLVSPGYTHL 416

C.auris CBS10913 AYDDLQKMPLINQTIKETLRLHMPLHSIFRKVMNPLVVPNTKYVVPKGHYVMVSPGYAQT 411

C.parapsilosis ATCC22019 SYEDLQQMPLVNNTIKETLRLHMPLHSIFRKVVSPLVVPNTKYIVPRGHHVLVSPGYAHT 411

C.albicans SC5314 TYEDLQKLPSVNNTIKETLRMHMPLHSIFRKVTNPLRIPETNYIVPKGHYVLVSPGYAHT 411

C.tropicalis ATCC750 TYEDLQKLPLVNNTIKETLRMHMPLHSIFRKVMNPLRVPNTKYVIPKGHYVLVSAGYAHT 411

* ** * * ****** * **** **** * * * * * ** **

C.kefyr ATCC26548 QDRYFPNAKEFNPHRWDNDAASSY----ASGEQVDYGFGAISKGVSSPYLPFGGGRHRCV 471

C.kefyr ATCC28838 QDRYFPNAKEFNPHRWDNDAASSY----ASGEQVDYGFGAISKGVSSPYLPFGGGRHRCV 471

C.kefyr Kw135/15 QDRYFPNAKEFNPHRWDNDAASSY----ASGEQVDYGFGAISKGVSSPYLPFGGGRHRCV 471

C.kefyr Kw1672/11 QDRYFPNAKEFNPHRWDNDAASSY----ASGEQVDYGFGAISKGVSSPYLPFGGGRHRCV 471

C.kefyr Kw3153/14 QDRYFPNAKEFNPHRWDNDAASSY----ASGEQVDYGFGAISKGVSSPYLPFGGGRHRCV 471

C.kefyr Kw2153/18 QDRYFPNAKEFNPHRWDNDAASSY----ASGEQVDYGFGAISKGVSSPYLPFGGGRHRCV 471

C.kefyr Kw20-12/20 QDRYFPNAKEFNPHRWDNDAASSY----ASGEQVDYGFGAISKGVSSPYLPFGGGRHRCV 471

C.kefyr Kw196-11/20 QDRYFPNAKEFNPHRWDNDAASSY----ASGEQVDYGFGAISKGVSSPYLPFGGGRHRCV 471

C.kefyr Kw28-10/12 QDRYFPNAKEFNPHRWDNDAASSY----ASGEQVDYGFGAISKGVSSPYLPFGGGRHRCV 471

C.kefyr Kw823/14 QDRYFPNAKEFNPHRWDNDAASSY----ASGEQVDYGFGAISKGVSSPYLPFGGGRHRCV 471

C.kefyr Kw3619/13 QDRYFPNAKEFNPHRWDNDAASSY----ASGEQVDYGFGAISKGVSSPYLPFGGGRHRCV 471

C.glabrata CBS138 QEEFFPKPNEFNIHRWDGDAASSS---AAGGDEVDYGFGAISKGVSSPYLPFGGGRHRCI 473

C.auris CBS10913 NEKWFPRANEFDPHRWDEETSSN-----IDTDAVDYGFGKVTKGVSSPYLPFGGGRHRCI 466

C.parapsilosis ATCC22019 NERFYKDASDFNPHRWDESAST------NDAGEVDYGFGKVSKGVSSSYLPFGGGRHRCI 465

C.albicans SC5314 SERYFDNPEDFDPTRWDTAAAKANSVSFNSSDEVDYGFGKVSKGVSSPYLPFGGGRHRCI 471

C.tropicalis ATCC750 SDRWFEHPEHFNPRRWESDDTKASAVSFNSEDTVDYGFGKISKGVSSPYLPFGGGRHRCI 471

* ** ****** ***** ***********

C.kefyr ATCC26548 GEHFAYMQLGTLLSNFVRTLTWKYANDKDTVPVPDFQSMVTLPLAPGEIEWTLRK 526

C.kefyr ATCC28838 GEHFAYMQLGTLLSNFVRTLTWKYANDKDTVPVPDFQSMVTLPLAPGEIEWTLRK 526

C.kefyr Kw135/15 GEHFAYMQLGTLLSNFVRTLTWKYANDKDTVPVPDFQSMVTLPLAPGEIEWTLRK 526

C.kefyr Kw1672/11 GEHFAYMQLGTLLSNFVRTLTWKYANDKDTVPVPDFQSMVTLPLAPGEIEWTLRK 526

C.kefyr Kw3153/14 GEHFAYMQLGTLLSNFVRTLTWKYANDKDTVPVPDFQSMVTLPLAPGEIEWTLRK 526

C.kefyr Kw2153/18 GEHFAYMQLGTLLSNFVRTLTWKYANDKDTVPVPDFQSMVTLPLAPGEIEWTLRK 526

C.kefyr Kw20-12/20 GEHFAYMQLGTLLSNFVRTLTWKYANDKDTVPVPDFQSMVTLPLAPGEIEWTLRK 526

C.kefyr Kw196-11/20 GEHFAYMQLGTLLSNFVRTLTWKYANDKDTVPVPDFQSMVTLPLAPGEIEWTLRK 526

C.kefyr Kw28-10/12 GEHFAYMQLGTLLSNFVRTLTWKYANDKDTVPVPDFQSMVTLPLAPGEIEWTLRK 526

C.kefyr Kw823/14 GEHFAYMQLGTLLSNFVRTLTWKYANDKDTVPVPDFQSMVTLPLAPGEIEWTLRK 526

C.kefyr Kw3619/13 GEHFAYMQLGTLLSNFVRTLTWKYANDKDTVPVPDFQSMVTLPLAPGEIEWTLRK 526

C.glabrata CBS138 GELFAYCQLGVLMSIFIRTMKWRYPTEGETVPPSDFTSMVTLPTAPAKIYWEKRHPEQKY 533

C.auris CBS10913 GEQFAYVQLGTILATYVYNIKWRFKKD-GSLPPVDYQSMVTLPMEPAEIEWEKRETCVY 524

C.parapsilosis ATCC22019 GEQFAYVQLGTILTTFVYNLKWKLAN--GKVPDVDYTSMVTLPQHPAEIVWEKRDTCVI 522

C.albicans SC5314 GEQFAYVQLGTILTTFVYNLRWTIDG--YKVPDPDYSSMVVLPTEPAEIIWEKRETCMF 528

C.tropicalis ATCC750 GEQFAYVQLGTILTTYIYNFKWRLNG--DKVPDVDYQSMVTLPLEPAEIVWEKRDTCMV 528

** *** *** * * * *** ** * * * *
